# Modifications in the Methods of Treating Transverse Fractures of the Patella*For the degree of Doctor in Medicine.

**Published:** 1886-05

**Authors:** George A. Staples

**Affiliations:** Dubuque, Iowa


AN INAUGURAL THESIS.*—SOME MODIFICATIONS TO THE ORDINARY METHODS OF TREATING TRANSVERSE FRACTURES OF THE PATELLA. *By* GEORGE A. STAPLES, A. M., M. D., *of Dubuque, Iowa.*

A communication made by M. Richelot† in 1883 to the Société de Chirurgie, of Paris, relative to the comparatively frequent casualties arising from treating transverse fractures of the patella by opening the knee joint and suturing the fragments, has awakened, especially among French surgeons, a reaction against this method of treatment, although it is advocated by the great surgeon Lister. In our own country Dr. J. A. Wyeth,‡ who has collated nineteen cases thus treated, with three deaths, has also protested against the operation; but it is probably true that the majority of Modern operators coincide with the views of Lister,§ as expressed in his paper

---

* For the degree of Doctor in Medicine.

†Contribution a l'etude des suites des fractures, de la rotule, etc. These de Paris, '84. Par H. Labouin.

‡Med. Record, 1882, No. 22, p. 596; and ibid., 1885, Nov. 21, page 581.

§London Lancet, Nov. 3, 1883.

before the London Medical Society, and regard the measures therein set forth as a great advance in surgery. Stimulated by the fact that three cases of transverse fracture of the patella have recently come under my observation, and noticing the conflicting views as regards treatment of that fracture, I was led to study somewhat the literature of the subject, and I present below the results of that study.

The causes of separation of the fragments in the fractures in question are, first, the action of the quadriceps extensor muscle, and, second, the effusion into the joint.

Gulliver* has shown that, when the tendinous expansion of the quadriceps covering the patella is torn, the fragments can be separated 4 2-10 lines. If the diastasis is greater, the fibrous capsule must be split on both sides. Malgaigne,* by experiments on cadavera, obtained like results, and Girdner† found that if he cut only the covering of the patella, a diastasis of no more than ½ to ¾ of an inch could be attained, but if he cut freely into the capsule the fragments could be pulled apart three inches.

If, now, a fracture occurs, and the fibrous covering and capsule of the patella be torn on both sides to a greater or less extent, the upper fragment is drawn up by a contraction of the quadriceps; it will also be drawn somewhat outward by the contraction of the vastus externus,‡ this muscle being relatively very strong. Retraction of the ligamentum patellæ will also pull the lower fragment somewhat downward.

As stated above, the second cause of the diastasis is intra-articular effusion. This fact was noted by Malgaigne§ and the

*A. Hentzolt. Ueber die Behandlung, der subcutanen Querfracturen der Patella, etc., p. 8.

†Hamilton. Fracture of the Patella, p. 90.

‡Harrison Allen. Human Anatomy, Section III., p. 308.

§Hentzolt, loc. cit.

English surgeons of the last century, and various means were adopted to get rid of the effusion. Flajani* in 1786 employed revulsives for that purpose. Thos. Castle† applied an evaporating lotion of white wash and used leeches till the tension subsided. Of late years cold and compression (Ravoth) and vesication (M. M. Guyon and Gosselin) have been strongly advocated.

The latest investigations of Riedel, and the clinical observations of Volkmann, Kocher and Langenbuch, show that absorption of this effusion does not take place quickly, and that retention of blood clots in the joint creates a chronic hydrarthrosis, that, in its turn, helps to keep the fragments asunder.

Riedel,‡ by experiments on dogs, found that complete absorption generally took place within two months. But in dogs the clotting of the effused blood does not seem to hinder the absorption materially, while the reverse is true in man. Langenbuch § did not succeed, moreover, in aspirating the hemarthrosis twenty-four hours after the injury, because the blood had already clotted. Lücke had the same experience. Secher and Volkmann,|| three days after the accident, found the blood already coagulated. If cases have been recorded where the effusion remained fluid from six to eight days, it should not be inferred that it will stay in this fluid state, for a part will certainly clot, and the clots may persist for a long time. Volkmann¶ observed fourteen weeks after an

*Du Traitement des Fractures Transversales de la rotule, etc. These de Paris, 1884. Par C. Divernesse.

†Castle. Manual of Surgery, Lord, 1834. p. 394.

‡Deutsche Zeitschrift Chir. Bd. XII.

§Langenbuch. Zehr. Congr. der Deutscher Gesellschaft fur Chirurgie.

||Volkmann. Centralblatt fur Chir. 1880. Nr. 10.

¶Hentzolt, loc. cit.

injury a large mass of clots remaining in the joint, and the same thing was seen by Hentzolt * at the expiration of a half year. Jarjavay, in 1861, first evacuated the effusion in the knee-joint. He was followed by Voillemier and Broca.* In 1872 M. Dubrueil* thus operated on a case and the operation was followed by death. This fact being reported to the Société de Chirurgie of Paris, that body condemned this therapeutic measure for the relief of the effusion.

In Germany, Volkmann† first made the articular puncture, removing the fluid two days later with a syringe. The fragments were thus drawn together by plaster strips and held by a plaster of Paris bandage. In eight weeks he thus obtained a strong bony or fibrous union.

Schede,‡ besides immediately aspirating the joint, syringed out the cavity with a three per cent. carbolic acid solution. The wound was then covered with protective silk, on which he placed salicylated cotton. Five cases were thus treated by him with an excellent result in each. Jourowski, in 1878, reported several cases in which good results were obtained by removal of the effusion. Marcy and Cok recommend the operation, but not the antiseptic injection; Kocher § is of the same opinion for the reason that phenic intoxication might occur.

M. Poinsot|| after gleaning from literature the accounts of nine cases in which the fluid was evacuated under antiseptic precautions (the fatal case reported by M. Dubrueil occurring

---

*Archives Generales de Medicine. Mars. 1884. Ad. Jalaguier, Revue Critique.

†Hentzolt, loc. cit.

‡Schede Centralblatt fur Chirurgie. 1877. p. 657.

§Kocher. Centralblatt fur Chirurgie. 1880.

||Poinsot. Revue de Chirurgie, 10 Janvier, 1882, p. 52.

anterior to the use of antiseptics), concludes that the procedure is comparatively harmless, and that, because of the ease with which it allows the fragments to be approximated, it should be employed as soon as possible after the injury. M. Jalaguier, in a very interesting article, remarks that since the work of M. Poinsot has been published, aspiration has steadily gained ground among French surgeons; and recently the Société de Chirurgie of Paris has publicly reversed its judgment of 1872. The change of opinion is well expressed by M. Trélat* in the following words: "To-day I puncture joints that are full of blood, and, nevertheless, in the discussion of 1872 I publicly combatted the practice, declaring that I would prefer a large opening of the joint. My change of opinion is the result of the introduction of the antiseptic method into surgical practice." In England, where arthrotomy with bony suture has aroused such important discussions, it seems that the question of puncture to evacuate the effusion has been left a little in the shade. The operation, though, in that country has its earnest partisans, particularly Mr. C. Heath† and Mr. Hutchinson, who regard the effusion as the chief cause of the diastasis.

Its strongest advocates are the German surgeons. Hentzolt says: "Experience to date speaks in every case in its favor, since this procedure, carried out with the strictest antiseptic precautions is completely harmless." Volkmann, Schede,‡ Secher, Rinne,§ and Lücke express similar opinions. Marcy‖ also supports the operation, basing his advocacy on a large

---

*Jalaguier, loc. cit.

†N. T. Med. Record, May 31, 1884.

‡Schede. Centralblatt fur Ch. 1877. No. 42.

§Rinne. Centralblatt fur Ch. 1877. No 49 and 50.

‖Marcy. Centralblatt fur Ch. 1880. No. 27.

experience; he operated 124 times upon 75 different knee joints, affected from the most varied causes, with but one bad result.

It seems fairly proven that puncture of the joint, properly carried out, is an innocent operation; since, therefore, it is assuredly undeniable that the persistence of a large quantity of blood in the synovial sac must exercise a harmful influence on the synoval membrane, and since, by the state of distension in which it places the joint, it cannot but hinder the approximation of the fragments, it appears the part of wisdom to evacuate the intra-articular effusion as soon as the patient is seen.

In the treatment of transverse fractures of the patella the custom has long been to flex the thigh upon the body and to extend the knee, thereby securing relaxation of the triceps. Valentin* first recognized this principle and proposed elevation of the extremity. Astley Cooper† proposed that the patient take a half-sitting posture with the foot elevated and leg extended. Langenbeck and others keep the patient in bed and let the extended limb lie flat on a pillow. Coster‡ placed the limb on a plane inclined from the foot to the trunk, so that the limb thus raised formed with the axis of the body an angle of about 45°. R. Liston§ recommends extension by a straight splint extending from a little below the tuberosity of the ischium below the middle of the leg. The method of Valentin, with slight flexion at the hip, is generally preferred, as it is more suitable for long use. An appropriate position in many cases is competent to effect firm union. It can certainly be competent if the lateral parts of the capsula are not

---

*Hentzolt, loc. cit. p. 14.

†A. Cooper. Treatise on Dislocations and Fractures. p. 207.

‡Coster. Manual of Surg. Operations. Phil., 1825.

§R. Liston. Elements of Surgery. Phil., 1837.

much torn, and there is little separation. It is better first to empty the effusion by aspiration. Prof. v. Wahl,* of Dorpat, in '83 treated in this way an old woman, 60 years of age, where the separation of the fragments was not great. The joint was twice punctured under the spray; the wound was irrigated with a bi-chloride of mercury solution, and dressed with iodoform dressings. Simple elevation of the limb was practiced. The result was perfect.

Warren,† of Boston, earnestly champions treatment by position, in the following words: "Transverse fracture of the patella, attended, as it often is, by great separation of the fragments, may be most successfully treated by position alone." He also believes that the limb does better lying flat in bed than when elevated.

In this connection the writer begs leave to report the following case. A boy, aged nineteen, while tending a saw in a saw-mill, was thrown violently, by the giving way of the machinery, against a heavy stick of timber. The accident occured Aug. 11, 1883. Two days later he was brought to Dubuque, when Dr. Jas. M. Boothby, was summoned. The writer, in company with the doctor, saw the patient. A fracture of the left patella, transversely, the fragments being separated somewhat over an inch, was discovered. A temporary dressing had been applied by a surgeon at the place where the patient had been injured.

The patient was at once placed in bed, his leg resting on an inclined plane. Little dressing, because of the tenderness of the joint, could be applied. The boy was discharged, well, on Oct. 10th, there then being but a slight fissure perceptible be-

*Hentzolt, loc. cit. p. 15.

†Warren. Surgical Observations. Boston. 1867. p. 322.

tween the fragments. He, when last seen, on Feb. 1, 1885, has perfect use of the limb.

Coaptation of the fragments may, broadly speaking, be obtained by the direct or indirect method. To the former plan belong the various kinds of sutures, and usually as a prelude to arthrotomy; to the latter plan belong the different means employed without penetrating the skin and the many apparatuses applied after a preliminary rupture of the integument. I purpose only to consider those forms of indirect coaptation that leave the skin intact.

Malgaigne divided the dressings known at his time into four groups: (*a*) those exercising circular pressure, and more or less surrounding the patella; (*b*) those exercising parallel pressure, operating upon each fragment, while working transversely upon the axis of the limb; (*c*) those exerting concentric pressure, pressing from above downwards and from below upwards upon the circumference of each fragment, and (*d*) those that press only upon the upper fragment. Malgaigne expressess his opinion of them all in these words: "If there is an incontrovertible fact in surgery, it is the incompetency of these (the dressings) in the attainment of a regular and firm union." He explains this by the fact that the patella has no prominences sufficient to furnish points of support to the dressings, and that it is difficult to press down the quadriceps and ligamentum patellæ enough to make two furrows in which the straps can rest. Even if the dressing has been applied firmly the fragments dip posteriorly and the fractured surfaces bulge forward from each other. The idea that the swelling of the joint militates against true approximation, and that the surgeon in some way should allay this, does not seem to have occurred to Malgaigne.

It is interesting to note that simple extension by means of gutter splints is recommended by Paul of Egina.*

These fractures were treated by circular pressure by Albucassis* toward the end of the fourth century. He applied a splint with a fenestrum for the patella. This was firmly held by a bandage. The splint was afterwards replaced by a ring of leather, and the leather in its turn by twisted wire. Parallel pressure was first made by Muschenbrok,† a mechanic of Leyden. His apparatus, as modified by Arnaud, consisted of a posterior hollow splint of tin, the sides of which were pierced with holes, and of two broad concave crescents that were placed, the one above, the other below the patella. The crescents were fastened to the posterior splint with screws.

Concentric pressure was first used by Lavanguyon about 1680. The apparatuses were modified by Ravaton and Petit.*

Pressure acting solely on the upper fragment was first employed by Petit about 1770.†

The method of Astley Cooper‡ was to well bandage the extremity, and just above and below the patella to take several circular turns; strong bands were fastened laterally to these turns and their free ends tied in such a manner that the fragments could be drawn together. Later, Cooper substituted leather rings for the bandage and straps, with buckles for the lateral bands. Unfortunately, a bulging for-

* Divermesse, loc. cit.

† Hentzolt, loc. cit.

‡ Cooper, loc. cit.

ward of the fractured surfaces could not be prevented by this dressing. Boyer attempted, therefore, to remedy this defect by direct pressure over the fracture, and constructed an apparatus of which Malgaigne said: "It unites great strength with great simplicity." Boyer placed the limb in a well padded wooden splint bearing nails opposite the joint; to these nails he attached straps extending over and under the patella; over the knee-cap he then laid a compress moistened with a resolving fluid, and confined it by a roller.

Sansom* applies long strips of plaster reaching from the middle of the thigh over the fracture to the middle of the leg, and just over the fracture he makes a loop of 8–10 inches. Rollers are then placed as braces to the plaster and the loop twisted by a stick.

Castle† recommends, after extension, that a well padded splint be applied with the patient in the sitting posture; that a leather strap be buckled around the thigh, above the fracture, and from this that another strap be passed beneath the foot, the latter being raised. The strap is then brought up on the other side of the knee and buckled to the circular strap above the joint.

Woodman‡ employes two gutta percha splints, that, while yet warm, are fitted anteriorly and posteriorly. After they have hardened he cuts a fenestrum for the patella in the anterior splint. Rollers are then passed around both.

The dressings of Burge,§ Lonsdale,|| Lansdale,|| Beach,|| Pelikan|| and Wechler|| are similar in principle to the appa-

* Hentzolt, loc. cit.

† Castle, loc. cit.

‡ Woodman, Medical Times and Gazette, Aug. 20, 1870.

§ N. Y. Med. Record, Apr. 15, 1868.

|| Hentzolt, loc. cit., p. 21.

ratus of Muschenbook, and exclude bandages. Burge applies well fitting sole leather splints, to which are attached extension weights. Pelikan uses pads above and below the patella, and a third of circular shape to press down the fragments from above.

It is unnecessary to go through the whole list of known dressings, some of them seeming to be of little value. It is of slight importance of what material the dressing consists, or whether, instead of the two concave plates of the Arnaud apparatus, smaller concentric or horse-shoe-shaped plates are chosen, and whether these are padded or not.

Immovable dressings, such as the starched bandage of Sentin, the dextrine bandage of Velpeau, or that of liquid silex were, earlier in the century, approved by many. Of late years plaster of Paris is much used, but it can only bring about a good result if the intra-articular effusion is removed (Heath). If this is not done the dressing must be often changed,—an unpleasant operation.

Much ingenuity has been exercised by American surgeons in the treatment of this fracture; the dressings of Agnew,* Hamilton and others are well known and popular. The plan of Prof. Hamilton,† approved by Prof. Gross,‡ as it was employed in the two cases described below, that came under my own observation, deserves especial notice.

It consists of a single inclined plane, long enough to support the thigh and leg, and about six inches wider than the limb at the knee. The plane is well padded and the member laid thereon, care being taken that the space under

---

* Agnew, Surgery, vol. I., p. 974.

† Hamilton, Fractures and Dislocations, p. 447.

‡ Gross, System of Surgery, vol. II, p. 211.

the knee is well filled. Then a sort of testudo genu with a figure of eight plaster bandage and roller is placed over the patella, first approximating the fragments as well as possible. Finally the whole limb is fastened to the splint by a second roller, reaching from the ankle to the groin. In the cases to be described, the only modification made was that the posterior splint was somewhat guttered and a light anterior one was added.

On July 13th, 1881, P. K., a brakeman, while getting on a train moving at the rate of ten miles an hour, struck his right patella on a bolt while the leg was somewhat flexed. He said that there was a loud snap and he felt as if shot. As he recovered himself he could feel that the knee-cap was injured, as, to use his language, "the upper piece stood out straight." Being a vigorous man, about twenty years old, he walked a short distance, dragging the injured member along. A temporary dressing was applied by a surgeon at Lansing, Iowa. The next day the patient was brought to Dubuque, where he was seen by Dr. G. M. Staples and the writer. The Hamilton dressing modified, as spoken of above, was put on, the fragments being separated one and a half inches. He was kept in bed and the wound regularly dressed. On Nov. 1st, patient went to work again, there being but a slight furrow noticeable. Flexion and extension were not good, and if the patient slipped slightly he noticed that the joint became painful and swollen.

On June 13th, 1883, while the patient was getting off a slowly moving car he stepped on a piece of wood and turned his right foot. He felt the patella snap and was at once disabled. When the writer saw him there was considerable swelling of the joint and the fragments were separated at least two inches. The same treatment was given him as before, and without any noteworthy trouble he could get about on Oct. 1st. His leg, though, was very weak, for he could not walk a long distance. For a short walk the leg was strong enough. There was a diastasis of about half an inch. In December of 1883, while descending some steps, the patient slipped and fractured the patella a third time, the separation being about one and a half inches. He did not call a surgeon, but dressed it himself and kept quiet but a short time. As the leg became useless he came under the charge of Dr. G. M. Staples, who kept the patient in bed for a month with the Hamilton dressing, and then freshened the edges of the patella fragments with a drill pressed through the integument. This was done on May 7th, 1884. The patient got about for the first time on July 28th, with the leg stronger than it has been since the first injury.

Whenever the patient slips, the leg is apt to be strained, and swell and be tender for a few days. He can now flex the leg to about a right angle with the thigh, and extend it fairly well. The knee cannot endure the cold, and he protects it with a silk cap and chamois skin. The diastasis has increased, being 2¾ inches when the limb is extended, and 4 inches when it it flexed. But the member seems to increase in usefulness every day. There is some muscular atrophy. Two inches below the patella the circumference of the right leg is 14 inches, of the left 14⅛ inches; four inches above the patella the circumference of the right leg is 15 inches, that of the left leg 15¾ inches.

I cannot find in literature any cases exactly similar to the above. At the Société Medicale de Reims, Séance du 16 Janvier, 1884, M. Langlet* showed a patient who had twice

*Union Medicale et Scientifique du Nord-Est., 15 Mai, 1884.

fractured his patella, the second time evidently by muscular action. The two fragments were separated the breadth of four fingers, but the patient could use the limb well if the knee was first bandaged.

A man, F. H., forty years of age, while passing over a frozen surface, slipped and fell, striking his right knee. He was brought disabled to the office of Dr. G. M. Staples, where he was seen by the writer. There was a transverse fracture of the patella, the fragments being separated about an inch. The accident occurred on Dec. 31, 1884. The leg was dressed as in the former case, and the patient sent home; he was then placed on his back in bed and the limb elevated. On Jan. 3, 1885, the wound was dressed, and atrophy of the quadriceps determined. Four inches above the patella the right leg measured 15¾ inches in circumference, the left at the same point 16 inches; while two inches below the patella the girth of the right leg was 12½ inches, that of the left 13 inches. The patient has to-day, March 31st, 1886, regained power in the leg every week, there being slight atrophy of the quadriceps, and apparently a strong fibrous union.

In his recent work on surgery Prof. Agnew * gives three indications for treatment of transverse fracture of the patella: first, to place the limb in that position which will secure the most complete relaxation of the quadriceps; second, to bring together and so retain the parts of the fractured bone, and third, to prevent anchylosis. In the concluding portion of this paper I purpose to discuss another indication, seemingly of first importance, but which, so far as I know, has not been noticed by English or American surgeons. That indication is to so treat the quadriceps as to avoid atrophy and degeneration of its structure.

---

* Agnew, loc. cit.

There is a certain amount of impotence that regularly follows these fractures; in some cases this seems due to a stiffness of the joint, in other cases to a certain flabbiness of the member. The latter condition is most frequent, and therefore, most important. It will be found that the patient cannot walk well, but is easily fatigued. Whether this state is due to the nature of the callus, to the length of the callus, or to muscular atrophy, is a most interesting and important question. Mobility of the fragments, want of nutritive elements in the lower fragment, the presence of synovial fluid mixing with the exudate of the callus, and poor coaptation have each been urged as a reason for non-bony union. Each probably has some influence, and, as the experience of Lister has shown, defective coaptation plays a considerable part. But as the following case proves, the chances of a bony union are as good with a simple dressing as with section or the hooks of Malgaigne.

In June, 1883, a patient entered the service of M. Lancereaux,* at la Pitié, for pulmonary tuberculosis; while there he fell upon the floor and fractured his patella. M. Gilsen, interne, applied a posterior splint and bandaged the extremity. On the fifteenth day the dressing was taken off and the patient left in bed. Five months later he died of phthisis.

At the autopsy the patella was found with its volume and dimensions normal. Two transverse fissures marked the spots where two fractures had occurred. There was a bony callus so firm that the point of a bistoury broke against it. M. Verneuil presented the specimen to the Société de Chirurgie. But a fibrous callus, if short and thick, is as good as a bony one; certain surgeons, according to Berger,† even prefer it, dreading the anchylosis that may come with a bony union.

---

* Labonne, loc. cit.

† Berger. Dict. Encycloped. des Sciences Med., T. V., p. 295.

Therefore, non-bony union cannot explain the impotence of the member, and the question is natural, why two patients with union of the fragments apparently similar, as has been noted, get about, nevertheless, in a very different way.

Valtât* some time ago remarked that muscular atrophy is provoked by arthritis; but in connection with fractures of the patella it has been but recently noted. In 1880 and 1881 M. Verneuil gave several clinical lectures on the subject at la Pitié. Before his time the impotence of the leg had been ascribed to the separation of the fragments.

Nélaton, in 1844, stated that the impotence was proportionate to the length of the fibrous ligament between the fragments. Malgaigne, in 1848, published a similar view.

Follin, in 1868, attributed the difficulty of locomotion to weakness of the extensors, the fibrous callus not offering the same leverage that a bony one would.

M. Gosselin† speaks several times of the loss in volume of the thigh muscles after fracture of the patella, as, he says, occurs in most muscles after fractures. But agreeing with Nélaton and Malgaigne, he ascribes the impotence to fibrous union or a long callus.

M. P. Berger‡ repeats the same doctrines. He says that the weakness of the leg is the result of persistent separation of the fragments over 1 ctm.; that the loss of power indicates the loss or partial abolition of the function of the quadriceps, but it is because the muscle cannot act efficiently upon the fragment of the patella.

On the other hand, M. Verneuil thinks that the chief factor in loss of power of the limb is atrophy of the quad-

* Labonne, loc. cit.

† Clinique Chir.—Gosselin.

‡ Berger, loc. cit.

riceps, particularly of the middle portion, the rectus femoris. Whether arthritis is present or not, atrophy comes on, usually very soon ; two days after the injury it may be determined by measurement, comparing the one with the other limb. Flaccidity and softness of the tissues on palpation also are quickly found.

It has been stated above that atrophy usually occurs after arthritis. Fracture of the patella is almost always followed by a more or less marked arthritis. But there are cases where that complication is wanting, or at least not evident, as in fractures from muscular action, and yet the atrophy of the thigh and leg muscles comes on as in cases of manifest arthritis.

M. Richelot* thus expresses himself :

"In weakening the member and abolishing its functions another factor, atrophy of the triceps (quadriceps), comes in. The muscle has indeed been seen to atrophy, and it has been said that because of defective union its degeneration impedes locomotion ; but the true rôle of that degeneration has been misunderstood, for the reason that fractures of the patella, and their consequences, were studied long before the influence of diseases of the joints upon the neighboring muscles was known. To-day we know that an arthritis of the knee, slight or severe, is accompanied almost inevitably by atrophy of the quadriceps, showing itself in the first few days. The arthritis appears to be the result of a reflex pathological action. The lesion may be ephemeral, or last some time, and get well spontaneously by exercise of the leg. Sometimes, however, it is rebellious and persistent, and that without any relation between the gravity of the inflammation and that of the atrophy being established. But a fracture of the patella cannot happen without articular

* Richelot, Union Medicale, etc., 2nd Sept., 1882.

inflammation of variable intensity, and this cannot escape the laws that govern arthritis evoked by other causes.

"If I am not deceived, the lesion of which I speak is better fitted, with its varieties, than any other to explain the functional states so diversified that are left after fractures of the knee-pan. The patient gets about well when the atrophy is very little and disappears promptly. When it is more severe, and gets well of itself in a few months, the functions of a member, believed to be forever compromised by a vicious union, are re-established little by little against every hope, and in spite of the persistence of a pronounced separation."

In support of the above statements M. Richelot showed two patients. Placing his hand upon the hip of the first of these he asked him to extend his leg ; the patient could not do so, no contraction even with his greatest effort took place, and none was transmitted to the upper fragment, which would have happened if the diastasis had been the peccant cause. M. Richelot, moreover, demonstrated that the circumference of the sound thigh was 5 centimeters larger than that of the injured one, while there was but a slight diastasis of the fragments.

In the second patient exhibited, the fragments were separated a good inch ; the interposed callus was apparently of slight thickness and strength, for a deep fossa existed. It seemed as though the action of the rectus would scarcely be transmitted to the patellar ligament, and yet extension was easily made. The patient could readily raise his heel from the bed, and the wounded member was scarcely less vigorous than the sound one. When the hand was placed on the quadriceps, energetic contraction was felt when he was bidden to extend the leg, and the most careful measurement could not find a difference of 1 ctm. in the circumference of the two thighs. Muscular atrophy, therefore, hardly existed.

M. Richelot therefore concludes that ingenuity employed to get the fragments in perfect coaptation is misspent, and that most of the manifold contrivances for that purpose should be laid to rest in the arsenal of surgery. To obviate the separation the most simple means are the best, and when after treatment of 30 to 40 days there is found a fibrous callus and movable fragments it is useless to prolong further repose and to condemn the patient to new attempts at coaptation. Attention must be given to the quadriceps, for when it regains its power the patient is cured.

The pathogeny of this atrophy is doubtful. Its cause is probably the arthritis, though M. Picqué* has collated cases of atrophy where marked arthritis did not exist. Whatever the cause, its seat is in the quadriceps, the rectus being first involved, and the rest of the muscle being gradually invaded. Faradic contractility is rapidly lost, either partially or entirely.

There is visible withering even to the eye, as observed in many cases by M. Petit. M. Labonne believes that this atrophy is the cause of the functional impotence that usually follows fractures of the patella, for the following reason: If union by fibrous callus leaves the leg impotent, and if at the same time muscular atrophy exists, it is difficult to tell the part that each plays. But if one of these two factors can be eliminated, and if the impotence disappears or persists, the true cause can be determined by induction. I cite a case in point.

*Picque. Reflexions Cliniques sur les fractures anciennes de la rotule.

A patient who had suffered from a fracture of the patella entered the service of M. Guyon. There was a fibrous callus 4 ctm. in length. Functional impotence was complete and considerable atrophy of the triceps with paralysis of that muscle was found. The muscle was treated by electricity for two months; in the beginning it did not contract at all under the influence of the current, but little by little contractions appeared, became more vigorous, and finally the patient could walk without a limp. Nay, more, the callus at first 4 ctm. in length, stretched to 6 ctm, though the cure was permanent. This case appears to demonstrate that the callus had nothing to do with the functional trouble and that its lengthening did not increase the infirmity so soon as the muscle had recovered its power. M. Larger* exhibited a patient with a callus 12 ctm. long who could walk almost without limping.

M. Picqué gives four observations of these fractures in laboring men with considerable diastasis in each. In two of these there was no atrophy of the quadriceps, in the two others a very slight withering of that muscle. They all had good use of the limb.

M. Guyon† had a case under his care where there was a diastasis of the flexed fragments of 7 ctm., with impotence of the member, that recovered perfectly under treatment by electricity. M. Labonne has collated 13 cases markedly similar to the above.

M. Badin,‡ reporting a fracture of the patella on his own person, stated that while the diastasis increased the func-

* Societe de Chirurgie de Paris. Nov. 1883.

† Labonne, loc. cit.

‡ Revue Medicale de Toulouse. Mai, 1884.

tions of the limb were regaining their power. Treatment by baths and massage had, in six weeks, reduced the difference in circumference between the healthy and injured thigh from 6 ctm. to nothing in some parts, to a single centimetre in others.

M. Guilliot,* commenting on another case in point, said: "That observation well shows how impotence is not in direct sequence to the separation of the fragments, but how it is due for the most part to atrophy of the quadriceps. In this patient the muscles of the thigh are generally little atrophied and still act in their movements by their insertions upon the inferior fragment of the patella and upon the tibia. This fact, then, endorses the opinions of MM. Richelot, Verneuil and Le Fort in the recent discussion in the Société de Chirurgie and condemns the practice of Lister, who makes primitive suture of the fragments."

In the case of P. K., described above, the patient stated that as the thigh gradually approached the other in size, although the separation continually became greater, the limb regained its power. In the case of F. H., the limb has also done well, there being little atrophy of the quadriceps. But the multiplication of examples, as have been given, though interesting, cannot make our case stronger. Besides causing impotence of the leg, M. Verneuil† accuses atrophy of the muscle of subjecting the patient to the danger of new fractures, either from falls or in the effort to avoid these. For when retraction has succeeded to atrophy the rectus becomes a solid, inextensible cord, capable of causing fracture easily. M. Labonne relates a case where the patient, suffering for two years from atrophy, fell to the ground without

* Societe Medicale de Reims-Seance du 16 Janvier, 1884.

† Societe de Chirurgie de Paris. Seance du 7 Nov., 1883.

striking his knee, and yet sustaining a transverse fracture of the patella.

As treatment for the atrophy, M. Verneuil advises, and has successfully employed, faradization. This is to be begun as soon as the traumatic pain has disappeared, and the fragments are maintained by a simple dressing. Each séance is to last five to ten minutes, and is to be given every two days. Faradization appears to act not only upon the muscle electrified, but even upon the nutrition of the member, as several observers, Prof. Le Fort among others, have noted. The muscles of the leg, as well as those of the thigh, even when only the triceps is thus treated, increase in size. When atrophy has existed a long time faradization is not efficient, and in such cases Dr. Ferras, of Paris,* employs with success the following means: a cold morning bath of twenty minutes, afternoon massage of the limb for half an hour, followed by a douche of luke-warm water.

### CONCLUSIONS.

The treatment of transverse fractures of the patella should not be addressed to approximation of the fragments alone—this is but one end among others to be attained. A simple apparatus, such as the Hamilton dressing, is preferably to be employed, since it performs the same service that the suture does without the dangers. It is always better to relieve the articular effusion, when attempting to reduce the diastasis, by vesication or aspiration.

Functional impotence after these fractures is due in general to premature atrophy† of the quadriceps. Atrophy

* *Revue Med.*, de Toulouse, Mai, 1884.

† At the first French Surgical Congress (Paris, April, 1885), M. Richelot, speaking from a further experience, reaffirmed his views as to the importance of the quadriceps muscle in fractures of the patella.

is constant, but varies in degree. When it is marked, it usually succeeds pronounced arthritis. This atrophy should be combatted by electricity from the beginning.

